# Supplementary material for: ‘We do not talk about it’: Engaging youth in Malawi to inform adaptation of a mental health literacy intervention
Source: PLoS One. 2022 Mar 29;17(3):e0265530. doi: 10.1371/journal.pone.0265530 (PMC8963557; doi:10.1371/journal.pone.0265530)
Supplement: S1 Fig — (DOCX) [file pone.0265530.s001.docx]

**Project title: Mental Health literacy project**

**Community youth stakeholder focus group questions**

| **Domain** | **Questions** |
| --- | --- |
| Introduction | My name is … and I am working on a project which aims to improve mental health literacy in Malawi particularly among young people and their peers.  There are no right or wrong answers to the questions asked. Everyone’s participation and views are important.  The discussion will be recorded so that we can accurately analyse the information we gain from these discussions. The recordings will be stored securely and kept confidential (only accessed by the study team).  Do I have everyone’s permission to start? |
| Description of population | Before we start our discussion, it would be good if you briefly introduced yourselves. Please say;   - Your name (you don’t have to use your real name) - What you do?   Who do you live with?  What does a typical day in your life look like?  What do you do in your pass time? |
| Priority health or social concerns | What are the most important issues affecting young people in Malawi at this time?  Which of these issues is of the greatest importance to you and your community / social circle?  How do these challenges affect the mental health of young people and those in their community? |
| Perceptions of substance use | Alongside mental health, recent data is showing an increase in harmful substance use like binge drinking, tobacco or chamba smoking in Malawi. There have also been reports of deaths due to overdosing on hard drugs.  In your view, do you think alcohol consumption or smoking is harmful?  When does substance use become harmful?  Can you think of reasons why people drink excessively or take more harmful substances?  Do you think there is a substance use problem in Malawi? If yes, why?  Are there particular people or groups in Malawi that you think commonly use substances in a harmful way or are at risk of substance use? If yes, why? |
| Changes since COVID-19 | How has coronavirus affected the concerns you mentioned earlier? (give examples of what has been mentioned)  How have priorities for young people’s health and social concerns changed since the pandemic?  How has the pandemic affected the mental health of young people in Malawi?  Have you been personally affected at all? |
| Perceptions of sources or access to mental health services | Where do Malawians most likely go when they need mental health information?  How about when they are mentally unwell?  What barriers do Malawians face when seeking mental health services? |
| Suggested strategies to increase mental health awareness | What kind of mental health services are needed in the community?  Do you think mental health education programs are needed? If so, what kind?  What strategies would be effective to deliver mental health information to the community?  How would you prefer to get information about mental health? What about substance use issues? |
| Suggested trusted credible sources of health information in the community | How do you guys typically obtain mental health information? (Probe: healthcare providers, family or friends, internet, etc?)  Who would you turn to if you needed information about mental health?  What people or sources are the most trusted and credible? |
| Suggested locations for mental health support services/ awareness programs | What would be good places to provide mental health awareness programs or support services for young people in Malawi? |
| Conclusion | Anything else you would like to add that you think hasn’t been covered in this discussion?  Thank you for your time. |
