# Supplementary material for: ‘We do not talk about it’: Engaging youth in Malawi to inform adaptation of a mental health literacy intervention
Source: PLoS One. 2022 Mar 29;17(3):e0265530. doi: 10.1371/journal.pone.0265530 (PMC8963557; doi:10.1371/journal.pone.0265530)
Supplement: Attachment — Submitted filename: PONE-D-21-29945 response to reviewers 3.2.22.docx [file pone.0265530.s002.docx]

To: Dr Emily Chenette

Editor-in-chief, *PLOS One*

3^rd^ February 2022

Dear Dr Chenette and associated PLOS One editors

**RE: Manuscript submission reference PONE-D-21-29945**

Many thanks for the opportunity to revise our manuscript. You will find attached clean and track change revisions of our manuscript, “**‘We do not talk about it’: Engaging youth in Malawi to inform adaptation of a mental health literacy intervention”**. We indicate below how we have addressed comments from reviewer #1.

- Thank you for giving me the opportunity to review this manuscript. It is very interesting, and quite up to the date. I have some comments and doubts for the authors.

Thank you for your positive feedback, acknowledging interest of this paper, especially how it addresses current issues regarding youth mental health in Malawi. We have addressed your comments below and revised our manuscript accordingly

- First of all, the population interviewed are universitary people, why?

Thank you for your question. The population interviewed was not just university people. As outlined in the abstract and the ‘study setting’ paragraph under the ‘Materials and Methods’ section, the sample included young people from universities and other community settings e.g. youth organisations in Malawi (see page 6 line 3-9).

- Refereing to the sample, the authors said that 76 people agreed to participate; 44 of them enroled into the interviews, 25 did not give an answer and 6 had connectivity problems... That sum reaches to 75 people. There ir one missing person.

Thank you for noting this typing error. It was 75 people who agreed to participate initially. This has been amended on the manuscript under the ‘participant recruitment’ section (page 6 line 25)

- How did the authors coded the topics and the subtopics? Which was the followed procedure?

We used qualitative content analysis as an approach to analysis data from the focus group transcripts. The procedure followed to code the themes and subthemes is detailed in the ‘Data collection, analysis, and management’ section. We have revised this section with more details and an additional Table 3 (see page 9) to clarify this process (see page 8 line 13 to page 9 line 21). We have also included a paragraph on rigor of the data analysis process (page 10 line 5 to page 11 line 13)

- It would have been really interesting to also know the proposals of the youth in regard to mental health improvement, in order to stablish future research lines.

Thank you. This has already been outlined in the results under the section ‘suggested mental health solutions’ (see page 27 line 5 to page 28 line 20), where the youth proposed several ideas regarding mental health improvement. Examples in this section include more awareness raising of mental health, having a mental health practitioner within schools for consistent access to psychological support for students, creating local youth-friendly mental health services and top-down mental health advocacy strategy from government to facilitate mental health literacy in communities countrywide

- What are the main objectives of these results obtained? Which is the next step related to the improvement of youth Malawi's mental health?

Thank you for your comment. We have added details on work we have done and related next steps to the discussion section (see page 31 line 7-24)

We have also addressed additional comments from the editor below:

1. We have revised headings, tables and figures labels in the manuscript in line with the PLOS ONE style templates found at
<https://journals.plos.org/plosone/s/file?id=wjVg/PLOSOne_formatting_sample_main_body.pdf> and
 <https://journals.plos.org/plosone/s/file?id=ba62/PLOSOne_formatting_sample_title_authors_affiliations.pdf>

2. In line with PLOS ONE data availability guidance, we have uploaded the study’s focus group discussion guide as the minimal anonymized data set to facilitate replication of our study findings as a supporting Information file (S1 Fig on page 33 line 18). Please update our Data Availability statement on our behalf to reflect the information we have provided.

3. We have uploaded our 2 figure files to the Preflight Analysis and Conversion Engine (PACE) digital diagnostic tool and inspected them for image clarity and content.

Addressing the above comments has improved our manuscript and we are confident that the paper now meets PLOS ONE’s publication criteria. We look forward to your favourable opinion on our revised manuscript.

Yours sincerely

Dr Sandra Jumbe
