# Supplementary material for: ‘We do not talk about it’: Engaging youth in Malawi to inform adaptation of a mental health literacy intervention
Source: PLoS One. 2022 Mar 29;17(3):e0265530. doi: 10.1371/journal.pone.0265530 (PMC8963557; doi:10.1371/journal.pone.0265530)
Supplement: Attachment — Submitted filename: PONE-D-21-29945R1 response to reviewer 2.3.22.docx [file pone.0265530.s003.docx]

To: Dr Emily Chenette

Editor-in-chief, *PLOS One*

2^nd^ March 2022

Dear Dr Chenette and associated PLOS One editors

**RE: Manuscript submission reference PONE-D-21-29945R1**

Many thanks for the opportunity to revise our manuscript. You will find attached clean and track change revisions of our manuscript, “**‘We do not talk about it’: Engaging youth in Malawi to inform adaptation of a mental health literacy intervention.”** We indicate below how we have addressed the comment from reviewer #1.

- Thank you for consider my previous comments.
   The only issue still concerns me is related to qualitative statistical analysis. Now is more complete and clear, but, did the authors use any statistical program to conduct these analyses, such as Atlas.ti or similar? If not, why?
   Thank you for your effort and your job.

Thank you for acknowledging our efforts in addressing the original comments from reviewers to our manuscript. We are glad that you feel the manuscript is now complete and clear. Regarding your question, we already stated in the manuscript on page 8 lines 12-15 that we used NVivo to conduct our qualitative analysis. We have added a description of what NVivo is on page 8 lines 15-17 to make this aspect clearer.

Addressing the above comment has further clarified the data analysis section of our manuscript and we are confident that the paper now meets PLOS ONE’s publication criteria. We look forward to your favourable opinion on our revised manuscript.

Yours sincerely

Dr Sandra Jumbe
